# Prognostic Impacts of Age, Diagnosis Time, and Relapses in Primary CNS Lymphoma

**DOI:** 10.3390/jcm13164745

**Published:** 2024-08-13

**Authors:** Sona Ohanyan, Chen Buxbaum, Polina Stein, Shimrit Ringelstein-Harlev, Shahar Shelly

**Affiliations:** 1 Department of Neurology, Rambam Medical Center, Haifa 3655306, Israel; ohanyan@ualberta.ca (S.O.); c_buxbaum@rambam.health.gov.il (C.B.); 2 Department of Neurology, University of Alberta Hospital, Edmonton, AB T6G 2B7, Canada; 3 Neuroimmunology Laboratory, Ruth, and Bruce Rapaport Faculty of Medicine, Technion—Israel Institute of Technology, Haifa 3525408, Israel; p_stein@rambam.health.gov.il; 4 Department of Hematology and Stem Cell Transplantation, Rambam Health Care Campus, Haifa 3109601, Israel; s_harlev@rambam.health.gov.il; 5 Department of Neurology, Mayo Clinic, Rochester, MN 55905, USA

**Keywords:** primary central nervous system lymphoma, early diagnosis, mortality, age, prognosis

## Abstract

**Background:** The incidence of lymphomatous involvement of the central nervous system (CNS) has been increasing in recent years. However, the rarity of the disease has resulted in a scarcity of available data regarding its clinical presentation, natural history, and prognosis. We aimed to investigate the neurological characteristics of uncommon lymphomatous involvements confined to the CNS and to identify key variables that could serve as predictive biomarkers for treatment outcomes. **Methods:** We identified patients presenting with neurological symptoms and diagnosed with CNS-restricted lymphomatous involvement between 2005 and 2023. **Results:** We identified 44 cases, 93% of which were diagnosed with primary central nervous system lymphoma (PCNSL) and 7% with intravascular lymphoma. The median time from symptom onset to diagnosis was 47 days (range: 6–573 days), with no statistically significant difference between patients older and younger than 60 years (*p* = 0.22). The median follow-up time was 1144 days (range: 27–3501 days). Cognitive deterioration was the most common presenting symptom, occurring in 19 out of 44 patients (43%). Brain MRI revealed that lobar lesions were the most frequent location of lesions, found in 24 out of 44 patients (55%). By the end of the study period, 30 patients (68%) had died, with a median survival of 666 days (range: 17–3291 days). Death was significantly more common in patients who experienced relapses (p = 0.04; 95% CI: 0.99–0.03), with these patients having a four times higher chance of death (HR = 4.1; 95% CI: 1.01–16.09). The time to diagnosis significantly correlated with survival (*p* = 0.02; 95% CI: 0.005–0.54), as did the Eastern Cooperative Oncology Group (ECOG) performance status at the last follow-up (*p* = 0.006; 95% CI: 0.0012–0.62). Patients aged over 60 years did not exhibit a higher likelihood of death (*p* = 0.19; HR = 2.3; 95% CI: 0.63–8.61); however, the threshold age at diagnosis for the maximally predicted mortality was 64 years (ROC = 0.73; *p* = 0.03). **Conclusions:** Patients had significant delays in diagnosis, affecting patient outcomes. Cognitive deterioration and lobar lesions were prominent clinical and radiological features. Mortality was notably higher in patients with relapses and those who had a longer time to diagnosis.

## 1. Introduction

Primary central nervous system lymphoma (PCNSL), a rare and aggressive form of non-Hodgkin lymphoma (NHL) that affects the brain, poses a significant diagnostic challenge, especially due to its diverse clinical presentations [1]. Diffuse large B-cell lymphoma (DLBCL) is the most common histology in PCNSL and occurs in 90% of cases, with the rest being T-cell lymphoma, Burkitt’s lymphoma, and low-grade lymphomas [2]. In recent years, PCNSL has shown a notable increase in incidence, reaching 0.5 per 100,000 people in immunocompetent patients [3]. Over the last three decades, significant progress in treatments and prognosis achieved through high-quality retrospective series, single-arm phase II trials, and a series of randomized clinical trials, all of which have collectively contributed to the establishment of treatment guidelines [4]. A rarer entity within this context is IVL, where the lymphoma is confined to the blood vessels within the CNS. The incidence of IVL is extremely rare, estimated at 0.001 per 1,000,000 per year [5]. It can affect any organ in the body but most commonly involves the CNS and is seen in 48% of all IVL cases [6].

The clinical presentations are heterogeneous, ranging from cognitive alterations, to headaches, focal neurological deficits, and seizures [7]. Additionally, constitutional symptoms, such as fever, malaise, weight loss, night sweats, and fatigue [8], may further cloud the clinical picture [4,9,10]. Historically, the prognosis for PCNSL has been dismal, typified by an untreated survival span of 1.5 months with a 5-year survival rate of 30% [10,11]. The landscape has evolved with the advent of combined modality therapies, incorporating high-dose methotrexate-based chemotherapy and whole-brain radiation therapy. Nonetheless, survival remains influenced by multifaceted determinants, the most important of which are age at the time of diagnosis, performance status, disease extent, and molecular tumor characteristics [8]. Notably, age over 60, an ECOG performance status exceeding two, and leptomeningeal involvement independently correlate with shorter survival [12]. Other aggressive forms of NHL can intricately involve the CNS. For example, IVL may present acutely, with both clinical and pathological manifestations resulting from cerebral blood vessel involvement [13]. In such cases, prompt diagnosis proves pivotal, as the time to diagnosis and initiation of treatment significantly influences prognosis [1]. Delay in diagnosis frequently attributed to the puzzling and nonspecific clinical features of PCNSL, such as headaches and cognitive decline.

This study aimed to describe the real-life prognostic effects of variables, specifically patient age at the time of diagnosis. We confined our cohort to individuals whose sole primary manifestation was neurological and comprehensively investigated the spectrum of clinical characteristics for this distinctive cohort.

## 2. Materials and Methods

This study received approval from the Institutional Review Board (IRB) or the Ethical Committee at Rambam Medical Center (RMC). We retrospectively identified all patients diagnosed with PCNSL in our oncology department, for whom clinical information was available at our center from 1 January 2005 to 3 June 2023. The clinical data retrieved using an electronic record retrieval system that captured all newly diagnosed cases of PCNSL. 

To ensure comprehensive data collection, we established a secure website-hosted database containing demographic information, details on comorbidities, clinical characteristics, radiological findings, diagnostic procedures, treatment modalities, treatment response, side effects, and instances of relapse. The quality of the database was maintained through a rigorous process: all information entered was independently reviewed by both a research technician and a neuro-oncologist, who cross-checked the data against each patient’s medical records. 

Patients enrolled in the present study had to meet the following criteria: (1) diagnosis of lymphoma confirmed by pathological or cytological (cerebrospinal fluid (CSF) or vitreous biopsy) examination; (2) negative findings in a full-body CT scan or FDG-PET scan; (3) age over 18 years; and (4) immunocompetence and a negative HIV status. Patients diagnosed with primary vitreoretinal lymphoma were excluded from this study.

### 2.1. Endpoints and Statistical Considerations

The tumor response was assessed according to the International PCNSL Collaborative Group criteria [2]. The ECOG performance status scale was used to assess physical outcomes. The ECOG performance status scale [14] uses a 5-point score to assess performance status (PS) using an ordinal 5-point scoring system that measures oncological disability. The ECOG performance status scale was calculated at two time points: before the first treatment and at the last follow-up. This study also included oncological and neurological outcomes, which were documented based on the latest medical records. These outcomes encompassed cancer status and neurological symptomatology, as evaluated by the attending physicians. Mortality and causes of mortality were reported based on data extracted from medical records.

### 2.2. Statistical Analysis

Group differences with ages (above and below age 60 years) were established using the Wilcoxon rank-sum test, also known as the Mann–Whitney U test. This test was chosen for our analysis to compare continuous variables that did not follow a normal distribution. The chi-square test was used for comparing categorical variables. Survival was analyzed using Kaplan–Meier and Cox regression analysis following the further grouping of our patients into those who died or survived. *p* < 0.05 was regarded as statistically significant.

### 2.3. Full Data Access Statements

The authors take full responsibility for the data, the analyses and interpretations, and the conduct of this research; they have full access to all the data; and they have the right to publish all the data. Anonymized data not published within this article will be made available upon request from any qualified investigator.

## 3. Results

### 3.1. Clinical and Demographic Factors

We identified 44 cases with primary lymphoproliferative process and neurological presentation, 41 of whom had a histopathological diagnosis of PCNSL and 3 of IVL, between 1 January 2005 and 3 June 2023. Of these cases, 22 were male. The median age at the time of diagnosis was 64 (range: 29–81, Figure 1), with 28 patients diagnosed at an age older than 60 years. The overall median time from symptom onset to diagnosis was 47 days (range: 6–573 days), with no statistical difference between patients younger and older than 60 years (*p* = 0.22). All patients underwent histopathological diagnosis via brain biopsy (42 cases) or vitreous body biopsy (2 cases, with both vitreous and CNS biopsies), complemented by CSF analysis. The median follow-up time was 1144 days (range: 27–3501 days). The clinical presentation exhibited a varied array of neurological manifestations, as detailed in Table 1. Specifically, cognitive complaints were reported in 43% (19/44) of cases, ataxia in 30% (13/44), and dizziness in 25% (11/44). Additionally, a range of other neurological symptoms were noted, including speech disturbances, muscle weakness, visual field defects or unspecified visual disturbances, headaches, epileptic seizures, and nausea and vomiting in one case.

### 3.2. CSF, Histopathological Laboratory, and Radiological Evaluations

CSF studies were conducted in 30 patients, with a median time from symptom onset to lumbar puncture (LP) of 48 days (range: 5 to 563 days). In most cases, there was an observed elevation in CSF protein levels (24/30), with a median CSF protein level of 62 mg/dL (range: 22 to 458 mg/dL). Lymphocytic pleocytosis was present in nearly half of the patients (14/30), with a median of 3 cells/mm3 (range: 0–120 cells/mm^3^). CSF cytological evaluation showed abnormalities in only 10% of patients (3/30). Repeat CSF testing in seven patients did not improve sensitivity.

Six patients experienced deep vein thrombosis (DVT), resulting in consequential pulmonary embolism in two cases. Among patients with DVT, 5 (83%) died during the study period. Cytology revealed abnormal findings in two cases of DLBCL and one case of non-differentiated lymphoma.

PET-CT or total-body CT scans were performed on all patients, confirming no evidence of extracranial involvement. Brain magnetic resonance imaging (MRI) was conducted in all but three cases, where CT scans were used for brain biopsy due to MRI contraindications.

Most patients displayed lobar lesions in MRI (24 cases, Figure 2), while other locations included the cerebellum (6), hypophysis (2), ocular bulb (2), hypothalamus (1), intraventricular (1), basal ganglia (8), leptomeninges (1), and periventricular regions (8). Notably, seven patients had brainstem lymphoma, and three exhibited IVL, all showing a stroke-like appearance in MRI scans.

Among the PCNSL patients, 11 had a primary infiltrating appearance, 8 showed ring-enhancing lesions, and 5 had hemorrhagic involvement, Figure 3. Histopathological examination revealed that 42 patients had findings consistent with DLBCL, with 1 case each of Burkitt-like lymphoma and non-differentiated lymphoma.

At the time of diagnosis, serum lactate dehydrogenase (LDH) elevation was observed in 41% (14 out of 34) of patients.

### 3.3. Comparison between IVL and PCNSL

A comparison between the IVL and PCNSL groups showed no differences in age at diagnosis (*p* = 0.2 (95% CI: 0.28–0.85)) or sex (*p* = 0.5 (95% CI: 0.006–0.54)). The time to histological diagnosis was relatively longer in the IVL group, with a median time of 192 days (range: 172–285) versus a median time of 38.5 days (range: 6–573) (*p* = 0.01 (95% CI: 0.03–0.98)). DVT was not statistically more common in the PCNSL group, with one case in the IVL group and five cases in the PCNSL group (*p* = 0.3 (95% CI: 0.3–0.95)). Cognitive symptoms were present in all three IVL patients (*p* = 0.03 (95% CI: 0.02–0.08)). CSF protein levels were not different between the groups (*p* = 0.3 (95% CI: 0.1–0.8)). Relapses were present only in the PCNSL group (26 cases) (*p* = 0.03 (95% CI: 0.01–0.09)). Death was more common in the PCNSL group, with 30 patients versus none in the IVL group (*p* = 0.008 (95% CI: 0.006–0.13)). The median follow-up time was 958.5 days in the PCNSL group versus 1794 days in the IVL group.

### 3.4. Chemotherapy, Radiotherapy, and Immunotherapy

Induction treatment with high-dose methotrexate (HD-MTX) was administered to 42 patients (95%), mostly as part of combination chemotherapy regimens. The median number of HD-MTX cycles was 6 (range: 1–10). Rituximab was given to 30 patients (68%), primarily after the year 2011, with a median of 4 treatments (range: 2–10). Intra-cerebrospinal fluid (CSF) therapy was administered to 34 patients (77%), with a median of 4 treatments (range: 1–10); the Ommaya device was used for this purpose in 2 patients (4.5%). Intraocular methotrexate therapy was provided to 4 patients (9%). Procarbazine was included in induction chemotherapy with HD-MTX in 40 patients (90%).

Treatment was prematurely stopped in 10 patients (22%), primarily due to treatment-induced renal, hepatic, and lung toxicities (1 patient), poor performance status (2 patients), hematological complications (4 patients), infections (3 patients), death (2 patients), and patient preference (1 patient).

Consolidation treatment was administered to 38 patients (95%). The reasons for avoiding consolidation included relapse (one patient) or were unknown (five patients). Cytarabine was given to 36 patients (81%), with a median of 4 courses, and vincristine was administered to 39 patients (88%), with a median of 5 courses (range: 1–10 courses).

Maintenance treatment was provided to 4 patients (9%), with 1 receiving temozolomide and 3 receiving other therapies (1 procarbazine and 2 HD-MTX). Treatment response and outcome data were available for all cases, showing overall deterioration. Stem cell transplantation was performed in 2 patients (4.5%) after relapse, both achieving complete response. A total of 8 patients (18%) received whole-brain radiation therapy (WBRT) during induction, with a median radiation dose of 41.4 Grey (range: 23–60 Grey). Salvage radiation therapy was administered to 12 patients (27%). For further treatment details, see Table 2.

### 3.5. Clinical Outcomes

The median overall follow-up period was 3.1 years (range: 27–3501 days). The median survival was 3.7 years (range: 599–2378 days; Figure 4). Among all patients, 59% (26/44) experienced relapse (26 in the PCNSL group versus 0 in the IVL group). Patients aged over 60 years did not exhibit a higher likelihood of death (*p* = 0.19; HR = 2.3; 95% CI: 0.63–8.61, Table S1) Similarly, differences in time to death (*p* = 0.25), serum LDH levels (*p* = 0.47), CSF protein levels (*p* = 0.06), time to lumbar puncture (*p* = 0.24), and functional status pre- and post-treatment (*p* = 0.06 and *p* = 0.09, respectively) were not associated with increased mortality.

Death occurred in 30 patients (68%) by the end of the study period, with a median of 666 days (1.8 years; range: 17–3291 days). Mortality was significantly more common in the group that experienced relapses (*p* = 0.04; 95% CI: 0.99–0.03). The causes of death among these patients included refractory lymphoma (13), treatment-related complications (1), ischemic stroke (2), sepsis (7), lymphoma relapse (2), unknown causes (4), and goals of care with hospice care (2). In total, 20 cases (46%) had more than 3 years of follow-up with repeated neurological evaluations.

The median number of relapses per patient during the follow-up period was 1 (range: 0 to 2 times), with a median time to relapse of 19.1 months (range: 22 to 3210 days). Six patients experienced relapses within 1 year from disease onset. The time from symptom onset to diagnosis appeared to be shorter in the group with no relapses (median: 31 days; range: 6–285 days) compared with those with relapses (median: 52 days; range: 6–573 days), though this difference was not statistically significant (*p* = 0.24).

At baseline, all 44 patients exhibited abnormal neurological examinations. Neurological functional outcomes, assessed at the first neurological visit post-treatment using the ECOG performance status, showed that 11 patients achieved normal physical function (ECOG = 0), 17 had abnormal neurological findings but were fully ambulatory (ECOG = 1), and 16 still required ambulatory aids (ECOG = 2, 3, and 4) (cane: 6; wheelchair: 4; bedridden: 6).

### 3.6. Prognostic Factors 

Age and time to diagnosis did not correlate with mortality (*p* = 0.05 (95% CI: 0.02–0.18)) but did with the time elapsed from symptoms to diagnosis (*p* = 0.04 (95% CI: 0.02–0.97)). Examining various age cutoffs for mortality among elderly patients—specifically those older than 60, older than 70, and older than 80 years—none of the cutoff ages reached statistical significance. Similarly, sex did not show a significant association with mortality (*p* = 0.51 (OR = 0.65; 95% CI: 0.18–2.35)) for 60 years (*p* = 0.89 (95% CI: 0.58–1)) and for ages older than 80 (*p* = 0.95 (95% CI: 0.69–1)). Patients who had relapses did show higher mortality (*p* = 0.04 (95% CI: 0.03–0.99)) with a hazard ratio of 4.1 (95% CI: 1.01–16.09). The threshold age at diagnosis for the maximally predicted mortality was 64 years (ROC = 0.73; *p* = 0.03). The time from symptoms to lumbar puncture did not significantly correlate with death (*p* = 0.23). Patients treated with anti-CD20 did not show differences in overall survival (*p* = 0.37 (95% CI: 0.18–0.81)) in our cohort. DVT showed no correlation with mortality (*p* = 0.36 (HR = 2.6; 95% CI: 0.27–24.64, Table S1)). The time to diagnosis significantly correlated with survival (*p* = 0.02 (95% CI: 0.005–0.54)) as well as the ECOG performance status at the last follow-up (*p* = 0.006 (95% CI: 0.0012–0.62)). 

Patients with relapses commonly presented with cognitive symptoms (*p* = 0.04 (95% CI: 0.04–0.98)) at the initial diagnosis. The time to relapse did not correlate with death (*p* = 0.32 (95% CI: 0.16–0.83)). We compared variables between the relapse and non-relapse groups and found no significant differences in age > 60 (*p* = 0.72), serum LDH levels (*p* = 0.19), CSF protein levels (*p* = 0.12), time to diagnosis (*p* = 0.35), or age at diagnosis (*p* = 0.29). Deep vein thrombosis (DVT) was also not more common in the relapse group (*p* = 0.64).

## 4. Discussion

**Figure 1:**
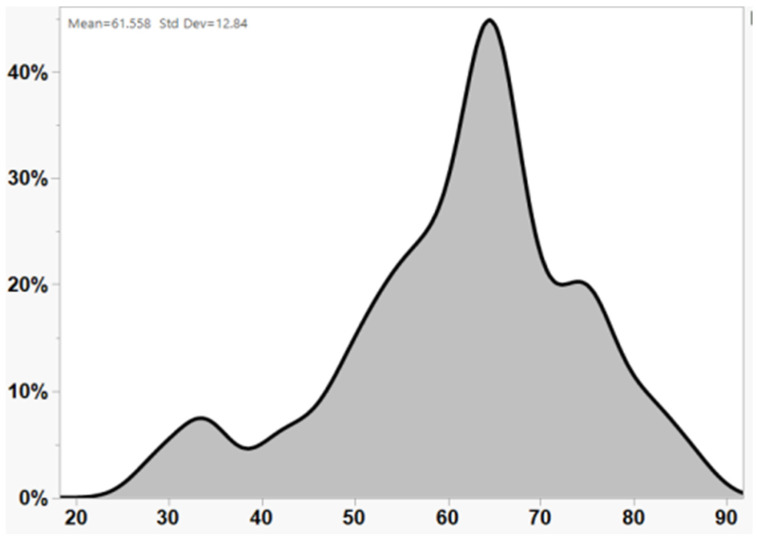
This figure illustrates the percentage of patients in various age groups within our cohort of primary CNS lymphoma patients. The x-axis represents the age in years, while the y-axis shows the percentage of patients. The shaded curve indicates the distribution, with the median age marked at 64 years.

**Figure 2:**
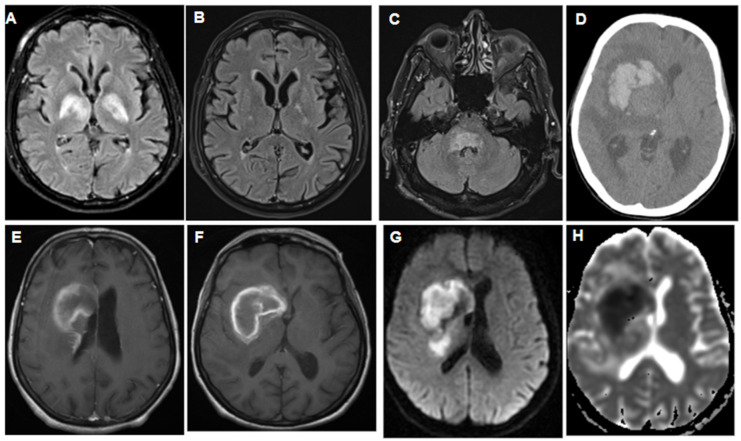
A series of imaging findings of patients with PCNSL and various neurological symptoms, demonstrating initial presentation, treatment response, and follow-up imaging, providing insights into the diagnostic process and radiographic treatment outcomes. (**A**): T2-flair MRI with gadolinium of a 79-year-old patient presenting with ataxia and episodes of delirium, showing bi-thalamic hyperintensity. (**B**): T2-flair MRI with gadolinium of the same patient after treatment, demonstrating evidence of near-complete resolution. (**C**): T2-flair MRI with gadolinium of a 64-year-old patient presenting with 6 months of ataxia and dizziness. (**D**): A CT scan of a 64-year-old patient with left hemi-syndrome, initially presenting as a hemorrhage. (**E**,**F**): T1+gadolinium MRI scan of the same patient exhibiting the same hemorrhage with contrast enhancement, as well as small periventricular gadolinium enhancement that was not visible in the earlier CT scan. (**G**): Diffusion-weighted imaging (DWI) of the hemorrhagic lesion, confirming the diagnosis of hemorrhagic stroke. (**H**): ADC (apparent diffusion coefficient) MRI series of the same patient, providing additional information about the hemorrhagic stroke.

**Figure 3:**
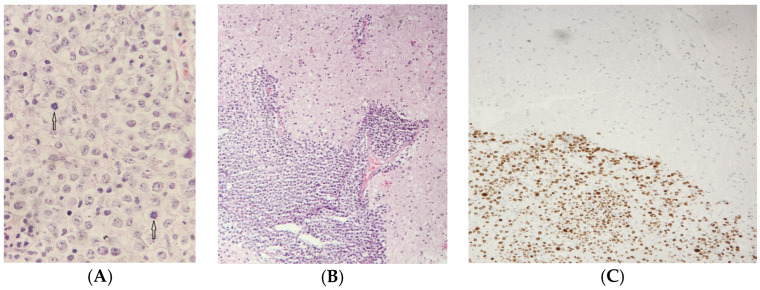
(**A**): Histological appearance of brain tissue showing diffuse infiltration by large, atypical cells. The tissue stained with hematoxylin and eosin at a magnification of 100×, large cells with prominent nuclear atypia are evident, along with numerous mitotic figures (indicated by arrows). (**B**): Immunoperoxidase staining at a magnification of 100× reveals a Ki67 proliferation index of approximately 85% in the large cells compared with the surrounding brain tissue. This indicates a high level of cellular proliferation. (**C**): At higher magnification (400×). The tissue is stained with hematoxylin and eosin.

**Figure 4:**
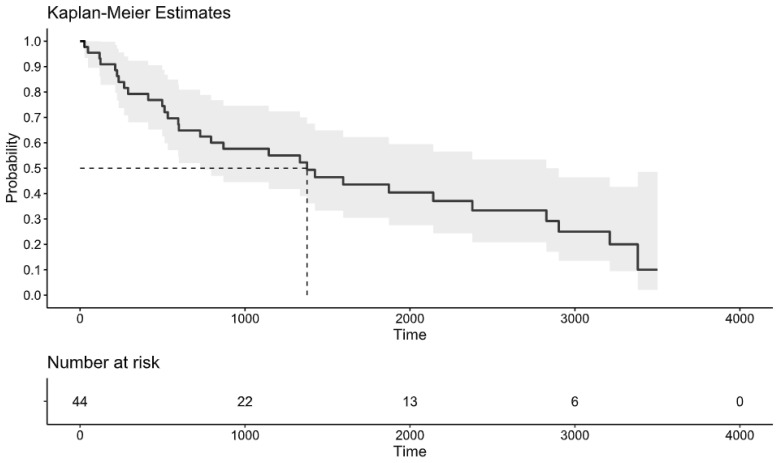
Kaplan–Meier survival analysis illustrating the overall survival of patients diagnosed with PCNSL. The median survival time from symptom onset was 1376 days.

In this study, we focused on the influence of age at diagnosis as a prognostic marker for PCNSL. To comprehensively analyze the impacts of different ages, we divided the age at diagnosis into three groups. The median age at diagnosis was 64 years, with the majority of patients (63%) being older than 60 years, indicating a trend toward higher prevalence in the elderly population (Figure 1). This finding aligns with previous studies [15,16,17]. Age is widely accepted as a significant prognostic marker in many cancers, including PCNSL, alongside performance status [18]. However, recent large-scale studies in various cancers and advancements in biological treatments have shown that older patients sometimes exhibit better responses to treatments and have improved survival rates [3,4]. The controversy over age cutoffs persists, with 50 years being used in the prognostic score developed by researchers at Memorial Sloan Kettering Cancer Center (MSKCC) [19] and 60 years being used in the International Extranodal Lymphoma Study Group (IELSG) score [12]. A recent publication suggested that an alternative age cutoff of 80 enhances the prognostic power compared with the other scales [20]. In our cohort, approximately 75% of patients older than 60 years died during the study period. However, none of the age groups showed statistically significant associations with mortality. We found that patients with a delayed diagnosis or relapse had a higher risk of death. The time from symptom appearance to clinical diagnosis was two months in our study, reaffirming its validity as a prognostic biomarker [12]. Our findings suggest that shortening the pre-treatment evaluation timeframe and not withholding treatment from older patients could improve survival outcomes (Figure 4).

The delay in the diagnosis of PCNSL might stem from its nonspecific clinical presentation, characterized by a wide array of neurological manifestations, as shown in Table 1. The most common symptoms include progressive cognitive decline and ataxia. Although severe headaches, nausea, and vomiting are frequently reported in the literature [9,11], these symptoms were less common in our cohort, occurring in only one-third of the patients.

A comparison between the IVL and PCNSL groups revealed that the time to histological diagnosis was significantly longer for the IVL group. The median time to diagnosis for IVL was 192 days (range: 172–285) compared with 38.5 days (range: 6–573) for the PCNSL group (*p* = 0.01). There was no significant difference in CSF protein levels between the groups. We attribute the prolonged diagnosis time for IVL to its rarity and the occurrence of stroke-like episodes, which can hinder further investigations in the early stages. Recent publications suggest that performing random skin biopsies in patients without lymphadenopathy, splenomegaly, or bone marrow abnormalities can improve the time to diagnosis [21].

The incidence of PCNSL appears to be increasing, with 0.5:1,000,000 cases per year [22,23], as we have also shown in our cohort, with almost twice as many new cases in 2023 compared with previous years. Real-world and population-based studies are still lacking to allow a better understanding of how to predict outcomes and stratify patients clinically and for experimental trials. The wide range of survival for PCNSL patients in our cohort (months–years) emphasizes the need for valid prognostic markers and improved clinical suspicion.

In our study, the previously published cutoff age of 60 did not reach statistical significance. However, a threshold of 64 years at diagnosis was found to be the most predictive of mortality. Our findings indicate that patients with relapses had a higher mortality rate and were four times more likely to die (HR = 4.1). This highlights the urgent need to improve the efficacy and durability of first-line therapies.

We also examined various prognostic markers for survival and risk of relapse. Our analysis showed that patients presenting with cognitive complaints often had involvement of deep brain structures and exhibited lower survival rates (*p* = 0.03), along with a higher risk of relapse (*p* = 0.04). This may be due to the dual involvement of deep brain structures (such as the periventricular regions, basal ganglia, brainstem, and/or cerebellum) and meningeal spread, both of which are strategic sites for tumor dissemination. These findings align with previous studies that identified these regions as significant markers for prognosis [12].

We investigated the diagnostic utility of CSF testing as a factor contributing to delays in diagnosis. CSF studies are routine tests in evaluating chronic or subacute neurological presentations, such as cognitive complaints [24]. In our cohort, the most common CSF abnormality was elevated protein levels, present in 80% of cases, with a median CSF protein level of 62 mg/dL. Lymphocytic pleocytosis was observed in half of the patients, though the median cell count was low (3 cells/mm). The sensitivity of cytological analysis was very low. Other studies have shown that any CSF abnormality is present in 80% of PCNSL cases [25], with CSF flow cytometry analysis reported to have a variable sensitivity ranging from 2% to 16% [26]. A review of three large studies, including more than 150 PCNSL patients with leptomeningeal involvement, found CSF cytological pathology in only 16% of the patients [27,28,29]. In our cohort, CSF testing did not aid the diagnostic workup in 93% of patients, even when repeated in 16% of cases. The very low detection frequency by cytological testing in our study appears inconsistent, given the common periventricular location and high rate of leptomeningeal involvement. One likely explanation for this apparent contradiction may be the fragility of tumor cells in the CSF, which are susceptible to environmental changes after lumbar puncture and prior use of corticosteroids [30]. The ratio of false-negative results may be reduced by increasing the volume of the CSF specimen to be analyzed (to 10.5 mL) and by ensuring the immediate processing of the sample.

The overall prognosis for PCNSL in our study was poor, with a median survival of 3.7 years, aligning with what has been reported in the literature [5]. After relapse, the median survival in our cohort was 2 years, which was higher than previously reported. In our cohort, 26 out of 44 patients (59%) experienced relapses in the PCNSL group, while none did in the IVL group. By the end of this study, 68% of patients had died. We investigated various factors relevant to survival [12], but neither increased CSF protein levels nor blood LDH correlated with survival. However, a shorter time to diagnosis did correlate with reduced survival. While rituximab has been reported to improve outcomes [31], we did not observe this effect in our study, similar to findings from the phase III study by Bromberg et al. [32]. Currently, high-dose methotrexate (HD-MTX)-based chemotherapy is considered the cornerstone of PCNSL treatment. In our study, induction treatment with HD-MTX was given to 42 patients (95%), mostly as part of combination chemotherapy.

Our study had several limitations, mainly due to the inherent biases of a retrospective study. Missing data and loss at follow-up did not exceed 10% for most items. Data on toxicity, especially quality-of-life questionnaires, were lacking, which is particularly important in elderly patients. A longer follow-up will be necessary to describe the population of long-term survivors and late relapses. Moreover, while our study included only 44 patients with PCNSL, our findings are consistent with the existing literature and serve to confirm previous evidence.

## 5. Conclusions

In summary, PCNSL poses a significant diagnostic challenge due to its subacute presentation and non-localizing signs, often resulting in diagnostic delays that impact survival by prolonging the pre-treatment phase (Figure 4). Diagnosis is particularly challenging, with the most sensitive diagnostic tools being abnormal brain MRI and brain biopsy. CSF analysis had low sensitivity in our study for detecting abnormal cells and typically did not significantly contribute to diagnosis. Survival does not appear to be directly affected by age at symptom onset but is influenced by the time to treatment initiation, with the overall prognosis remaining poor. Given the rarity of PCNSL, long-term follow-up studies are crucial for understanding long-term outcomes, which will inform the development of optimal surveillance strategies and supportive care interventions. Although recent advances have improved our understanding of PCNSL, additional collaborative research is essential. This ongoing research will be critical for developing more effective diagnostic, therapeutic, and supportive care strategies for PCNSL patient.

## Figures and Tables

**Table 1:** Clinical signs at admission in 44 patients with lymphoma dichotomized by age.

Symptom	No. of Patients under 60 Years (%)	No. of Patients of 60 Years and Over (%)
Cognitive complaints	7 (16)	12 (27)
Ataxia	3 (7)	10 (23)
Weakness	2 (5)	10 (23)
Headache	5 (11)	6 (14)
Dizziness	4 (9)	7 (16)
Speech disturbances	2 (5)	4 (9)
Unspecified visual disturbances	1 (2)	3 (7)
Visual field defects	1 (2)	2 (5)
Seizures	1 (2)	1 (2)
Nausea and vomiting	1 (2)	0 (0)

**Table 2:** Summary of variables and demographic information.

Variable	No. (%)
Age <60 years >60 years	16 (36) 28 (64)
Sex Male Female	22 (50) 22 (50)
Duration of symptoms pre-diagnosis <6 weeks >6 weeks	17 (39) 27 (73)
Lesions Multiple Single	19 (44) 25 (56)
Surgery Complete resection Stereotactic biopsy Partial resection	4 (9) 37 (84) 1(2)
Postsurgical treatment Untreated Radiation therapy Chemotherapy Combined treatment	0 0 35 (80) 9 (20)
Radiation therapy Yes No	8 (18) 36 (82)
Chemotherapy regimens MPV MP R-MT R-MPV HIDAC MTX ARA-C MPV */HDMTX	39 (89) 1 (2) 1 (2) 3 (7) 36 (81) 2 (5) 1 (2) 1 (2)

* MPV: methotrexate, procarbazine, and vincristine; MP: methotrexate and procarbazine; R-MT: rituximab and methotrexate; R-MPV: rituximab, methotrexate, procarbazine, and vincristine; HIDAC: high-dose cytarabine; MTX: methotrexate; ARA-C: cytarabine (cytosine arabinoside); MPV/HDMTX: alternating rituximab, methotrexate, procarbazine, and high-dose methotrexate.

## Data Availability

Anonymized data not published within this article will be made available upon request from any qualified investigator.

## Supplementary Materials

The following supporting information can be downloaded at: https://www.mdpi.com/article/10.3390/jcm13164745/s1, Table S1: Cox Proportional Hazard Model Analysis of Prognostic Factors.
