# Supplementary material for: Prognostic Impacts of Age, Diagnosis Time, and Relapses in Primary CNS Lymphoma
Source: J Clin Med. 2024 Aug 13;13(16):4745. doi: 10.3390/jcm13164745 (PMC11355736; doi:10.3390/jcm13164745)
Supplement: Supplementary file 1 [file jcm-13-04745-s001.zip › jcm-3129957-supplementary.pdf]

pSupplementary Material Table S1

Cox Proportional Hazard Model Analysis of Prognostic Factors.

Variable	Hazard Ratio (HR)	95% CI	p-value
Age > 60	2.3	0.63 - 8.61	0.19
Time to death	-	-	0.25
Serum LDH levels	-	-	0.47
CSF protein levels	-	-	0.06
Time to lumbar puncture	-	-	0.24
Functional status pre-treatment	-	-	0.06
Functional status post-treatment	-	-	0.09
Relapse	4.1	1.01 - 16.09	0.04
Age at diagnosis threshold (64 years)	-	-	0.03
Anti-CD20 treatment	-	0.18 - 0.81	0.37
DVT	2.6	0.27 - 24.64	0.36
Time to diagnosis	-	0.005 - 0.54	0.02
ECOG performance status at last follow-up	-	0.0012 - 0.62	0.006
Cognitive symptoms at initial diagnosis	-	0.04 - 0.98	0.04
Time to relapse	-	0.16 - 0.83	0.32
